# Sex-Differences and Temporal Consistency in Stickleback Fish Boldness

**DOI:** 10.1371/journal.pone.0081116

**Published:** 2013-12-04

**Authors:** Andrew J. King, Ines Fürtbauer, Diamanto Mamuneas, Charlotte James, Andrea Manica

**Affiliations:** 1 Department of Biosciences, College of Science, Swansea University, Swansea, United Kingdom; 2 The Structure and Motion Laboratory, The Royal Veterinary College, Hatfield, Hertfordshire, United Kingdom; 3 Evolutionary Ecology Group, Department of Zoology, Cambridge University, Cambridge, United Kingdom; University of Arizona, United States of America

## Abstract

Behavioural traits that co-vary across contexts or situations often reflect fundamental trade-offs which individuals experience in different contexts (e.g. fitness trade-offs between exploration and predation risk). Since males tend to experience greater variance in reproductive success than females, there may be considerable fitness benefits associated with “bolder” behavioural types, but only recently have researchers begun to consider sex-specific and life-history strategies associated with these. Here we test the hypothesis that male three-spined sticklebacks (*Gasterosteus aculeatus*) show high risk but potentially high return behaviours compared to females. According to this hypothesis we predicted that male fish would show greater exploration of their environment in a foraging context, and be caught sooner by an experimenter than females. We found that the time fish spent out of cover exploring their environment was correlated over two days, and males spent significantly more time out of cover than females. Also, the order in which fish were net-caught from their holding aquarium by an experimenter prior to experiments was negatively correlated with the time spent out of cover during tests, and males tended to be caught sooner than females. Moreover, we found a positive correlation between the catch number prior to our experiments and nine months after, pointing towards consistent, long-term individual differences in behaviour.

## Introduction

Individuals that behave in a certain way through time or across situations can be said to show a “behavioural type” [1], [2]. If various behavioural types are present within a population, a “behavioural syndrome” (i.e. behavioural consistency both within and between individuals) occurs [2], [3], [4]. One of the best studied behavioural syndromes is the syndrome of “boldness”. Boldness is a statistical correlation between behaviours that relate to risk, often reflecting the degree to which individuals balance fundamental trade-offs between risk and return [5], [6], and which can constrain individuals’ ability to behave optimally in all situations [7], [8]. For example, bolder individuals may benefit in a foraging context [9], [10], but also experience higher risk of predation [11], [12], [13]. Such consistent, cross-context correlation in behaviour is particularly interesting from an evolutionary perspective, and has resulted in the burgeoning field of animal personality and the study of behavioural syndromes.

Whilst much work has been devoted to understanding the origin and evolutionary consequences of differences between sexes [14], [15], [16], [17], only recently have researchers begun to consider sex-specific strategies and life-history in studies of behavioural syndromes [18], [19], [20]. Since males tend to experience greater variance in reproductive success than females, there may be considerable fitness benefits associated with high levels of exploratory behaviour [21], [22] and achieving greater than average foraging success [23]. Together, this may drive males towards high-risk but potentially high-return behavioural types [15], [24]. There is accumulating evidence that this is the case. For instance, male guppies (*Poecilia reticulata*) and great tits (*Parus major*) are generally bolder than females [20], [25], [26], and risky male behaviours (independent of other sexually selected traits) have been shown to predict male mating success in the fiddler crab (*Uca mjoebergi*) [24]. Age and/or reproductive stage can also interact with sex differences in behaviour. In grey mouse lemurs (*Microcebus murinus*) males are bolder than females in open-field and novel-object tests but show a systematic variation in their responses with age; young males with low current but high expected future fitness are less bold than older males with high current fecundity [27]. Similarly, field crickets (*Gryllus campestris*) exhibit sex differences in the repeatability of boldness across metamorphosis. Boldness is repeatable across metamorphosis in females, but not in males, which become less bold with maturation, presumed to reflect the risk associated with calling for mates [28]. Sex differences in boldness (that are linked to life-history trade-offs) might therefore be widespread, but more studies are required [18].

In the three-spined stickleback, *Gasterosteus aculeatus*, successful breeding is preceded by intense male-male competition to establish a territory, building and maintaining a nest, and courting of females [29], [30], [31]. Post-mating males are required to provide energy-intensive parental care, guarding and fanning the nest and fry [32], [33]. Both pre- and post-mating behaviours therefore limit feeding opportunities [33], [34], [35] and there may be considerable fitness benefits to males achieving greater than average foraging success [23]. This pressure, in turn, may predispose males towards bolder behavioural types compared to females [13], [15].

Here, we test this hypothesis by assessing the proportion of time male and female sticklebacks spend out of cover in search of food (foraging context [e.g. 36]), and the order in which they are net-caught by an experimenter from their aquarium prior to these behavioural tests (predation context [e.g. 37]). Given that bolder individuals often benefit in a foraging context, but suffer higher risk of predation (see above), we first expected to see a negative correlation between the proportion of time fish spend out of cover and their catch number, i.e., fish that explore a lot would be caught sooner (prediction 1). Concerning sex differences, we predicted that males would be caught significantly sooner than females (prediction 2), and would spend more time out of cover exploring their environment than females (prediction 3). Finally, we also tested whether we would see any long-term consistency (nine months) in catch number, indicative of stable individual differences in behaviour (prediction 4).

## Methods

### Subjects and Housing

All animal care and experimental procedures described here were approved as non-regulatory procedures by the Ethics and Welfare Committee of the Royal Veterinary College, London (URN 2011 1084). Subjects (n = 48) were a laboratory population of three-spined sticklebacks (*Gasterosteus aculeatus*) originally caught using a sweep net from Histon and Swaffham Bulbeck areas of the River Cam, Cambridgeshire, UK. Three-spined sticklebacks are not an endangered or protected species and therefore no specific permissions were required to collect them. All fish were individually identifiable by Visible Implant Elastomer (VIE) tags (Northwest Marine Technologies), and had not taken part in any previous experiments. All fish were housed together in an aerated and filtered, gravel-lined aquarium (120×40×30 cm) with plastic plants, and were fed defrosted bloodworms (*Chironomid larvae*) daily. One week prior to, and throughout experiments, fish were housed in individual, transparent, gravel-lined 2.8 litre self-cleaning polycarbonate tanks within a ZAD Series Aquaneering (Aquaneering Incorporated) rack system. This ensured similar environmental conditions for all subjects (tank positions were rotated weekly), and, critically, allowed us to standardise feeding intake throughout, thus minimising any potential inter-individual differences in motivations to forage. Since we were interested in behavioural differences between sexes, independent of any physiological changes (e.g. hormonal changes during breeding) that can affect both male and female behaviour (Webster & Laland 2011), the fish were kept at 16°C and under a photoperiod of 8L:16D hours (light:dark) prior to, and during experiments. This ensured that males showed no breeding colouration or courtship behaviour, and thus, were assumed to be reproductively quiescent [38]. Subjects were sexed after completion of our experiments by increasing the temperature of their tanks to 20°C for three days, which induced male breeding colouration (Ward et al. 2004a). Our sample consisted of 30 females and 18 males.

### Catch Number

When transferring subjects to the individual tanks within our rack system, fish were net-caught one by one from their aquarium by AJK and assigned a catch number (i.e. 1 = fish that was caught first; 2 = fish that got caught second etc.). To minimise any fatigue/stress response, the net was dipped once and swept through the tank. If no fish was caught, the net was removed and, following a 10 second pause, the tank was swept again. Nine months after taking part in exploratory behaviour experiments, subjects were once again net-caught from their aquarium by a different experimenter (CJ), and N = 30 fish could be identified from our original sample based on their VIE tags. Together, this provided us with “catch number one” data which we used to test our prediction that males will be caught sooner than females (prediction 2), and “catch number two” which allowed us to additionally explore the long-term consistency in the order in which fish were caught (prediction 4).

### Exploratory Behaviour

**Figure 1:**
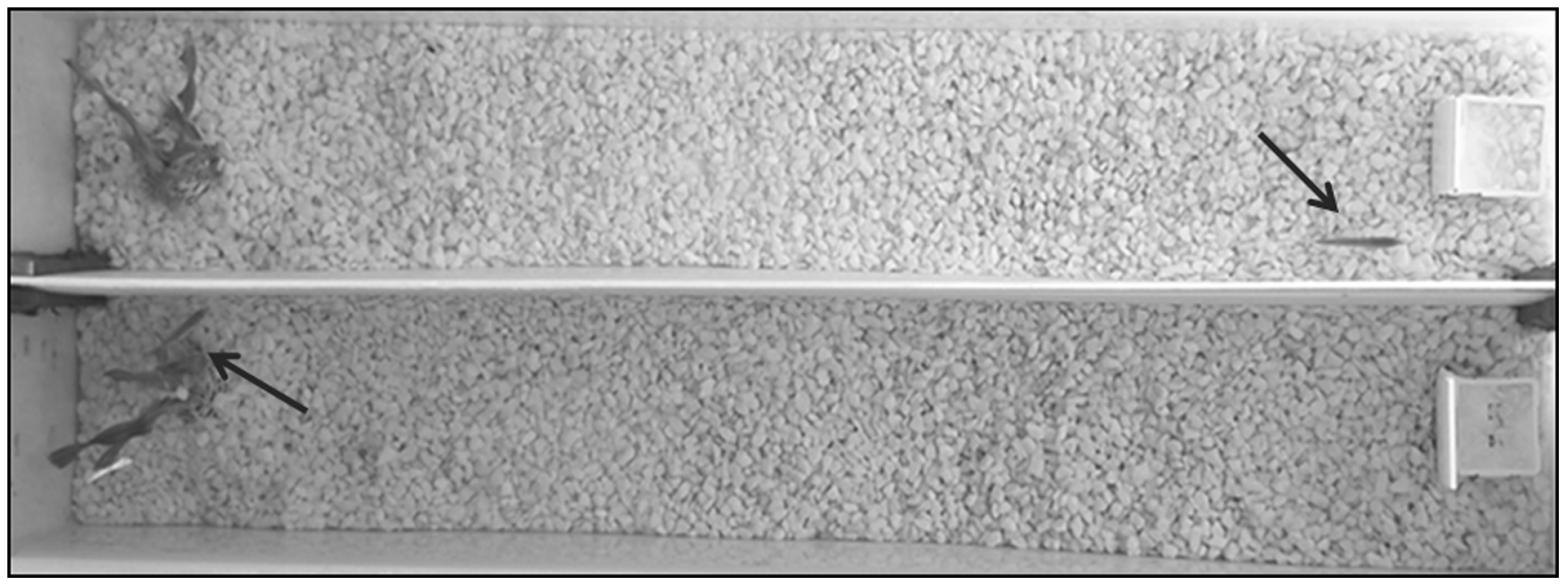
Exploratory behaviour assessment tanks. Fish are indicated by arrows. The fish in the upper lane is approaching the (empty) feeding tile on the right-hand side of the image, and the fish in the bottom lane is taking cover in the plastic plant located on the left-hand side. See methods for full description.

Tests of exploratory behaviour [following 36,39] took place in opaque test tanks placed within a 100×100×200 cm aluminium frame, enclosed by blinds on all sides to minimise the effects of external disturbances. When no tests were being conducted, the water was aerated using an air stone and air pump, and tanks were covered. Test tanks consisted of 15 cm wide lanes (Figure 1) lined with white gravel with a gradual slope in water depth: the deep end (15 cm) included a green plastic plant (identical to those used in their holding aquarium) to provide cover (Figure 1). The shallow end (4 cm) contained a small (2×5 cm) vertical white plastic screen behind which bloodworms could be placed. This meant that fish had to swim past the plastic screen in order to see the bloodworms when present.

Data was collected in cycles of four consecutive days, with fish spending one hour each day in the test tanks. On the first two days of a cycle, two bloodworms were placed behind the plastic screen and a single fish of known identity was introduced to the tank. Panasonic HDC-SD60 high definition video cameras were mounted directly above the tanks to record fish movement. Video was saved to memory cards, but also watched in real-time on a television in the lab which allowed us to observe whether the fish had found and consumed the bloodworm after 30 minutes. If the fish had eaten the bloodworms, they were replaced. Then, on days three and four of a cycle, no bloodworms were provided in the test tanks, and subjects were fed in their individual tanks later in the day. We therefore used the timing of transitions into and out of cover on days three and four to calculate percentage of time out of cover, and used this as our measure of the subjects’ tendency to explore the environment in search of food. A number of fish in our sample failed to locate and consume bloodworms over the one-hour period on one or both of the first two test days (n = 15; which were subsequently identified as 8 females, and 7 males). Since there may be individual, and/or sex differences in learning [40], [41] our main analysis of exploratory behaviour excluded those fish that failed to find the food, since they cannot necessarily be considered to be exploring in search of food on days three and four (we also conducted the same analyses on the full sample).

### Statistical Analyses

All data used in our statistical analyses are provided in File S1. Access to videos is available upon request to the corresponding author. We used Spearman’s rank correlations to test for consistency in the time fish spent out of cover on days three and four of our exploration tests, and to test for a correlation between the average time fish spent out of cover and their catch number (prediction 1). We used a Mann-Whitney-U test to test our prediction that males would spend more time out of cover in search of food than females (prediction 2). In each of these tests we used data from those fish that had located and consumed bloodworms on the first two days of our trials (n = 33 fish, see above), but also conducted statistical tests with our full sample of n = 48 fish. In order to test our prediction that males would be caught sooner than females (prediction 3), we used a Mann-Whitney-U test, using data for all fish (n = 48). Finally, to test for long-term consistency in catch number (prediction 4) we used a Spearman’s rank correlation. All tests were two tailed, and α was set at 0.05.

## Results

**Figure 2:**
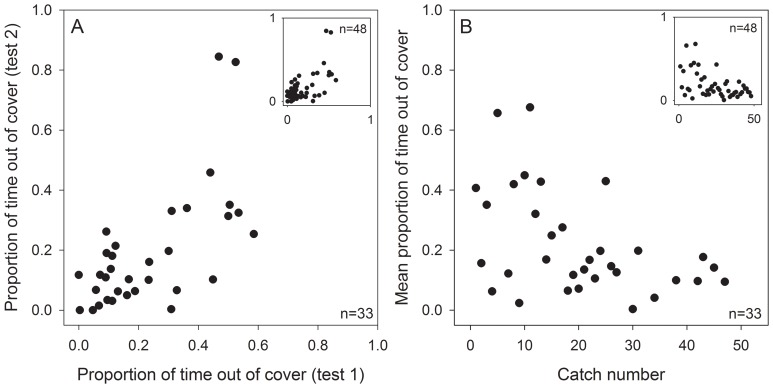
Exploratory behaviour and catch number. (A) Correlation between the proportion of time three-spined stickleback fish spent out of cover during two consecutive test days when placed in exploratory behaviour assessment tanks for one hour (Spearman’s ρ = 0.60, p<0.001, n = 33). (B) Correlation between the mean time spent out of cover and catch number, i.e., the sequence fish were net-caught from their holding tank (Spearman’s ρ = −0.39, p = 0.025, n = 33). Inset figures for both panels show data for the full sample (n = 48), including fish that failed to locate food on days one and two (see Methods for details).

The proportion of time individual fish spent out of cover in search of food was significantly positively correlated across our two test days (Spearman’s ρ = 0.60, p<0.001, n = 33; Figure 2A), and the average time fish spent out of cover was significantly negatively correlated with catch number (Spearman’s ρ = −0.39, p = 0.025, n = 33; Figure 2B) supporting our first prediction. These relationships hold for our full sample (n = 48 fish), including those individuals that failed to find food during days one and two of our exploratory behaviour experiments (repeatability of time out of cover: Spearman’s ρ = 0.50, p<0.001, n = 48; Figure 2A inset; correlation between time out of cover and catch number: Spearman’s ρ = −0.42, p<0.004, n = 48; Figure 2B inset).

**Figure 3:**
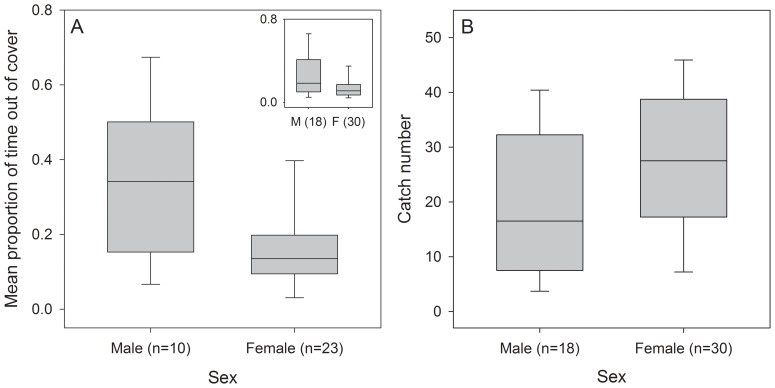
Sex differences in exploratory behaviour and catch number. (A) Box and whisker plots depicting sex differences in proportion of time three-spined stickleback fish spent out of cover (n = 10 males, 23 females). Inset figure shows data for the full sample (n = 48), including fish that failed to locate food on days one and two (see Methods for details). (B) Box and whisker plots depicting sex differences for catch number, i.e., the sequence subjects were net-caught from their holding aquarium (n = 18 males, 30 females).

Concerning sex differences, males spent more time out of cover than females (Mann-Whitney-U test; U = 56, p = 0.022, n = 33; full sample: U = 182, p = 0.062; Figure 3A), supporting our second prediction. Males also tended to be caught sooner than females, though this difference was not statistically significant (Mann-Whitney-U test: U = 185, p = 0.072, n = 48; Figure 3B), failing to fully support our third prediction.

**Figure 4:**
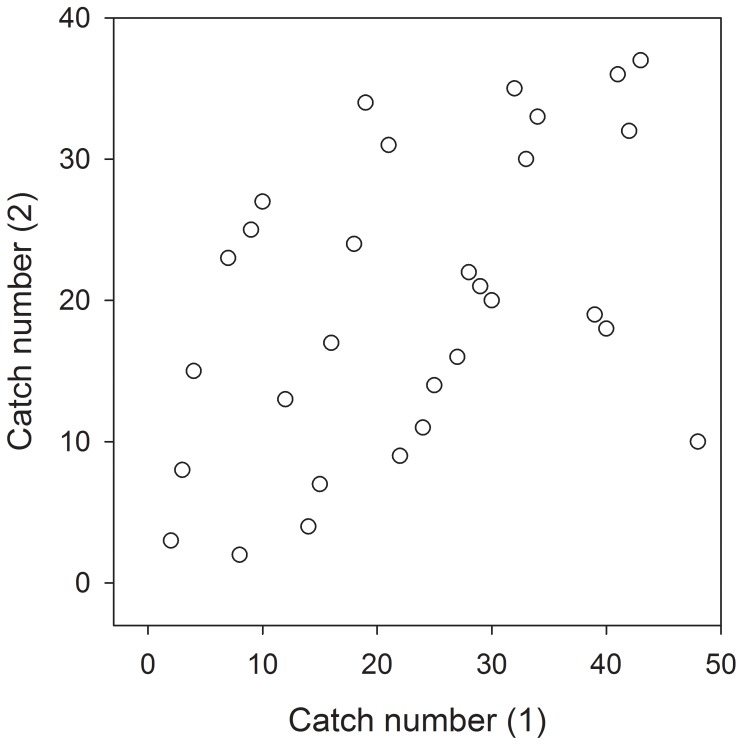
Consistent, long-term individual differences. Correlation between the first fish catch number (the sequence subjects were net-caught from their holding aquarium) and a second catch number for the same fish nine months later (Spearman’s ρ = 0.49, p = 0.006, n = 30).

Finally, the order in which fish were caught in our two capture events by different experimenters nine months apart was significantly positively correlated (Spearman’s ρ = 0.49; p = 0.006, n = 30; Figure 4), indicating long-term stability in the “catchability” of subjects supporting our fourth prediction.

## Discussion

Given that life-history theory predicts that differences in fitness expectations should result in systematic differences in risk-taking behaviour [42], we tested the hypothesis that males should tend towards high risk, potentially high return behavioural types [13], [15], as a potential consequence of specific pre- and post-mating behaviours that limit feeding opportunities [33], [34], [35]. Our findings support this hypothesis, and we showed that males spent more time out of cover exploring their environment in search of food than females, and showed a non-significant trend to be caught sooner than females by an experimenter. We also showed that the order in which fish were caught was stable across a significant portion of an individual’s lifetime (nine months) suggesting repeatability over long-time scales [43]. This is especially pertinent given that the catch-order protocol is difficult to standardise, our findings are based on a modest sample size, and repeatability estimates tend to be higher for studies conducted in the field compared to the laboratory and when the interval between observations is short [44].

Other studies on the same species have not found the sex differences in behaviour that we observe here [12], [45], and we consider three possible explanations for this. First, at a broad scale, there can be profound differences in behavioural types between populations [46]. Indeed, three-spined sticklebacks show considerable variation in behavioural syndromes at the population level [47], which might reflect different selection pressures, and, specifically, differences in predation risk across populations [48], [49]. Further investigation into such differences can be informative and may provide insight on the adaptive evolution of behaviour favouring ‘optimal’ trait combinations [36], [50].

Second, methods of animal capture [see 11,51], and a potential link between differences in exploratory behaviour and life span [52], [53] have the potential to affect variances observed in wild-caught laboratory subjects. For example, if bolder females were more prone to capture, this might reduce any sex-related differences tested for in the laboratory. Indeed, evidence for such personality-related sampling bias is accumulating, and is known to create bias in the traits we are interested in quantifying [54].

Third, dissimilarity in assays used across studies to test boldness might explain differences between our results and previous work, since it is seldom tested whether different assays designed to measure boldness are actually measuring the same trait in practice [55], [56]. We used an assay developed by Harcourt et al. [36], [39], [57] that involved a component of learning relating to the potential for a food reward. Since there may be individual, and/or sex differences in learning [40], [41] we excluded those fish that failed to find the food on our first two “training days”, as they cannot necessarily be considered to be exploring in search of food. However, even if we include these individuals in our analyses on the basis that they are expected to be “shyest” fish in our sample, our significant correlation between time out of cover and catch number stands (since only 2 of the 15 fish that failed to find food in our exploratory tests were in the upper 50% of our first catch number), and a trend for sex-differences in these responses persists. Thus, our assays appear to be measuring something meaningful about individuals’ tendencies to adopt high risk but potentially high return behaviours – the very traits we set out to measure.

Our results also provide a cautionary note for studies exploring how individual differences in behaviour can mediate dynamics of social interaction and group structure and functioning [58], [59]. For example, studies that explore how differences in behavioural types can affect social preference or social interaction often choose to study just one sex [60], or else do not report on sex differences [36], [57]. Consideration of one sex might therefore narrow the range of behavioural types considered (if sex differences exist), which would be problematic for experiments examining how the mix of behavioural types in a social group affects social dynamics. If sex is not considered when the composition of behavioural types within a group is manipulated (for example when it is not possible to sex subjects), it is possible that social interaction and group-level dynamics that are thought to emerge as a consequence of “personality” differences, could sometimes be more usefully understood as a consequence of sex differences. This is especially pertinent when one considers the mechanisms by which individuals may be able to identify bold or shy conspecifics versus male or female conspecifics [61].

Overall, this study adds to growing evidence demonstrating sex differences in boldness, providing further confirmation that sex can play an important role in shaping the behavioural syndromes we see in populations [27], [28], [62]. Furthermore, these individual differences in behaviour (especially variation among males) might play an important role in governing mating success [24]. Future work should now investigate the potential link between behavioural type and reproductive success for both male and female three-spined sticklebacks, since recent with the guppy (*Poecilia reticulata*) suggests that the combination of boldness characteristics within a pair can influence reproductive success [63].

## Supporting Information

File S1 **Supporting Information.** All data used in our statistical analyses and to produce the figures in the manuscript are labelled as follows: Fig 2A (main figure); Fig 2A (inset figure); Fig 2B (main figure); Fig 2B (inset figure); Fig 3A (main figure); Fig 3A (inset figure); Fig 3B; Fig 4.
(XLSX)
